# Antimicrobial resistance of *Salmonella* Indiana from retail chickens in China and emergence of an *mcr-1*-harboring isolate with concurrent resistance to ciprofloxacin, cefotaxime, and colistin

**DOI:** 10.3389/fmicb.2022.955827

**Published:** 2022-09-08

**Authors:** Yujie Hu, Yingying He, Scott V. Nguyen, Chang Liu, Chang Liu, Xin Gan, Wei Wang, Yinping Dong, Jin Xu, Fengqin Li, Séamus Fanning

**Affiliations:** ^1^NHC Key Laboratory of Food Safety Risk Assessment, China National Center for Food Safety Risk Assessment, Beijing, China; ^2^UCD-Centre for Food Safety, School of Public Health, Physiotherapy and Population Science, University College Dublin, Belfield, Dublin, Ireland; ^3^Public Health Laboratory, District of Columbia Department of Forensic Sciences, Washington, DC, United States; ^4^Department of Microbiological Laboratory Technology, School of Public Health, Cheeloo College of Medicine, Shandong University, Jinan, China; ^5^Food Science and Engineering College, Beijing University of Agriculture, Beijing, China; ^6^Institute for Global Food Security, School of Biological Sciences, Queen's University Belfast, Belfast, United Kingdom

**Keywords:** *Salmonella* Indiana, CIP-CTX co-resistance, resistance gene, poultry, colistin, plasmid, *mcr-1*

## Abstract

*Salmonella enterica* serotype Indiana (*S.* Indiana) in Chinese poultry meat has aroused widespread concern because of its high prevalence and strong antimicrobial resistance. In consideration of the relationship in our previous study between *S.* Indiana and co-resistance to ciprofloxacin and cefotaxime (CIP-CTX), which were the first-line drug which were used in *Salmonella* infection in clinical, the antimicrobial resistance (AMR) of 224 CIP-CTX co-resistant *S.* Indiana isolated from retail chicken samples in China were investigated, with the aim of characterizing the AMR profiles and related resistance mechanisms to ciprofloxacin and cefotaxime among these CIP-CTX co-resistant *S.* Indiana isolates, all of which showed multi-drug-resistant (MDR) phenotypes. GyrA (S83F and D87N/G) with ParC (T57S and S80R) were the dominant amino acid substitution types, with *oqxA*, *oqxB,* and *aac (6′)-Ib-cr* identified as common plasmid-mediated quinolone resistance (PMQR)-encoding genes. Five *bla*_CTX-M_ gene subtypes were identified with *bla*_CTX-M-65_ ranking at the top. Equally important, we obtained one isolate CFSA664 harboring the *mcr-1* gene was ESBL producer with co-resistance to nine in ten classes of tested drugs inclduing colistin. A single circular chromosome and 3 circular plasmids were found in its genome. Among the 26 AMR genes identified, 24 were located on plasmid pCFSA664-1, including three ESBL genes, while plasmid pCFSA664-3 owning only the *mcr-1* gene and sharing the same backbone structure with plasmids from Enterobacteriaceae. No insertion sequences were found near the *mcr-1* gene but a relaxase-encoding gene in the flank, which could transfer into *E. coli* J53 at a relatively high frequency. *S.* Indiana in this study exhibited highly drug-resistant phenotypes, contributing to the acceleration of the dissemination and emergence of this pathogen among different sources. Surveillance and a One Health strategy are needed to limit the emergence of *S.* Indiana along the food chain.

## Introduction

Poultry meat is known to be a major source of non-typhoidal *Salmonella* which causes human salmonellosis and ever-growing food safety concerns across this processing chain (Heredia et al., 2018). In total, there are currently over 2,600 serotypes of *Salmonella* as described by the World Health Organization (Issenhuth-Jeanjean et al., 2014; Thames et al., 2020). Many of the serovars associated with human infections are frequently found on broiler meats and three serotypes of *S. typhimurium*, *S. enteritidis*, and *S*. Heidelberg were the predominant (Foley et al., 2008; FSIS, 2020). Data reported that the prevalence levels in China differed from high to low among raw poultry meat, including chicken (26.4%), pigeon (22.6%), duck (10.1%), and other poultry meat (15.4%) and chilled poultry meat might be more likely to experience cross-contamination than non-chilled poultry meat in China (Sun et al., 2021). From the public health perspective, *Salmonella* contamination of poultry products has raised concerns, in particular in regard to the emergence and dissemination of the isolates resistant to fluoroquinolone and/or third-generation cephalosporin (e.g., ciprofloxacin and cefotaxime), the recommended first-line drug treatment for *Salmonella* infections (Antunes et al., 2016).

*Salmonella enterica* subsp. *enterica* serovar Indiana (*S.* Indiana) is one of the most common foodborne *Salmonella* serovars, being isolated from poultry meat (Gong et al., 2017; Hu et al., 2018a; Zhang et al., 2022). In recent years, *S.* Indiana, expressing a high level of antimicrobial resistance (AMR) to many common antimicrobial compounds, has been epidemiologically linked with an increasing tendency of infections (Hu et al., 2018b). It was reported that the high levels of multidrug resistance (MDR) of *S.* Indiana might be associated with the presence of a variety of mobile elements such as plasmids carrying complex resistance mechanisms and class 1 integrons (Gong et al., 2016). These features increase the risk of treatment failure in the clinical infections caused by serovars of *Salmonella*.

Following our previous surveillance on the quantitative prevalence of *Salmonella* contamination in raw whole chicken carcasses at the retail (Zhu et al., 2014; Hu et al., 2017, 2018a), we obtained a subset of 224 ciprofloxacin and cefotaxime (CIP-CTX) co-resistant *S.* Indiana isolates. This study aimed to sketch an overall view on AMR and related mechanism for CIP and CTX of this set of *S.* Indiana from chicken sources in parts of China, and to provide an insight into the genomic characteristics of a CIP-CTX co-resistant *S.* Indiana strain CFSA664, that expressed concurrent resistance to colistin mediated by *mcr-1* was also characterized at the meanwhile.

## Materials and methods

### Strains study collection

In our previous study (Hu et al., 2018a), 2,629 *Salmonella* were recovered from 1,680 retail whole chicken carcasses (including freshly slaughtered, chilled and frozen samples) between August 2010 and March 2012 at seven sites located in six provinces of China referring to North, Northeast, Northwest, South and East part of China. All strains were confirmed as belonging to the *Salmonella* genus and 463 (17.6%, 463/2629) of them were then identified as *S.* Indiana by serotyping, 224 of which were further identified as CIP-CTX co-resistant *S.* Indiana by antimicrobial susceptibility testing (AST), and these isolates were distributed as below: Beijing (*n* = 99); Jilin (*n* = 60); Jiangsu (*n* = 42); Shaanxi (*n* = 16); and Guangdong (*n* = 7).

### Antimicrobial susceptibility testing

A panel of fourteen antimicrobial agents, representing ten classes, were selected for these 224 selected CIP-CTX co-resistant *S.* Indiana isolates for AST by broth micro-dilution, the drugs included ampicillin (AMP), cefotaxime (CTX), ceftazidime (CAZ), chloramphenicol (CHL), ciprofloxacin (CIP), gentamicin (GEN), imipenem (IPM), nalidixic acid (NAL), ampicillin-sulbactam (SAM), trimethoprim-sulfamethoxazole (SXT), tetracycline (TET), meropenem (MEM), florfenicol (FFC), and colistin (CT). Minimal inhibitory concentration (MICs) value data for all compounds were recorded manually, and these data obtained were interpreted following the recommendations of CLSI (M100-S28 and M31-A3) and EUCAST (version 2018); extended-spectrum beta-lactamase (ESBL) production was then screened according to the protocol and breakpoints described in CLSI (M100-S28) as previously described (Hu et al., 2017). *Escherichia coli* ATCC 25922 and *Klebsiella pneumoniae* ATCC™700603 were included as quality control microorganisms in AST assays and ESBL confirmation, respectively.

### CIP and CTX resistance mechanism analysis

Molecular methods were used to determine AMR genes and to show the relevant resistance mechanism. Genomic DNA was extracted with a TIANamp bacterial DNA kit (DP302, Tiangen Biotech) from all isolates. PCR detections for both quinolone resistance determinant regions (QRDRs) including *gyrA*, *gyrB*, *parC,* and *parE* genes and PMQR-encoding genes including *qnrABCDS, qepA, oqxAB,* and *aac-(6′)-Ib-cr* were carried out according to the study reported previously (Ling et al., 2003; Park et al., 2006; Jacoby et al., 2008; Liu et al., 2008; Yamane et al., 2008; Cavaco et al., 2009; Kim et al., 2009; Bai et al., 2016). Additionally, two multiplex PCR reactions were used for the detection of ESBL genes including *bla*_CTX-M_ groups and subtyping was also performed based on the methods reported previously (Eckert et al., 2004; Xu et al., 2005).

### *mcr-1*–*mcr-10* gene screening

All 224 CIP-CTX-resistant *S.* Indiana isolates were screened to investigate the presence of *mcr-1*–*mcr-10* genes by multi-target PCR methods as previously reported (Rebelo et al., 2018; Borowiak et al., 2020; Lei et al., 2020). To achieve the aim of addressing the full resistance phenotypic pattern for any *mcr* gene positive *S.* Indiana isolate screened in this study, an extended AST was carried out, using additional selected antimicrobial agents relevant to Enterobacteriaceae, including 13 classes (composing 27 compounds) in total and all procedures of AST were processed according to the AST processing workflow described above.

### Whole-genome sequencing

A single colony of each CIP-CAZ-CT co-resistant isolate was cultured overnight in brain heart infusion broth (Beijing Landbridge, China) at 37°C. Genomic DNA was extracted and purified using a TIANamp Bacterial DNA extraction kit (DP302, TIANGEN BIOTECH, China) and then sequenced with the SMRT^®^ Pacific Biosciences (PacBio) RS II platform (Tianjin Biochip Corporation, Tianjin, China) with a 10-kbp template library preparation step with PacBio^®^ Template Prep Kit. SMRT Analysis v2.3.0 was used for *de novo* assembly according to RS Hierarchical Genome Assembly Process (HGAP) workflow v3.0. Subsequently, Consed version 28.0 was used to manually inspect and trim duplicate ends to generate single, complete, and closed sequences for each chromosome and plasmid. For data error correction, Pilon v1.23 was used with Illumina MiSeq sequencing read data, for which a library was prepared with a NEBNext^®^ Ultra DNA Library Prep Kit for Illumina (NEB#E7370) followed by sonication fragmentation (350 bp insert), and then loaded on the Illumina HiSeq platform with PE 150 sequencing strategy (Novogene, Beijing, China) using a HiSeq X Ten Reagent Kit v2.5 (Illumina, San Diego, CA). The closed genomes were deposited in National Center for Biotechnology Information (NCBI) and automatically annotated using the NCBI Prokaryotic Genomes Automatic Annotation Pipeline (PGAP, version 4.8).

### Bioinformatic analysis

The predicted serotype and multi-locus sequence typing (MLST) types were identified using the *Salmonella In Silico* Typing Resource (SISTR). Plasmid replicon types (Incompatibility groups or Inc. groups) and antimicrobial resistance genes were identified through the Center for Genomic Epidemiology (CGE) website with PlasmidFinder (v2.0) and ResFinder (v3.0), respectively. Virulence factors and related virulence genes were predicted with VFanalyzer of the VFDB (virulence factor database, current status of August 10, 2022). All genes, plasmids, and chromosome sequences used in this study were managed, aligned, and analyzed by Geneious prime (v2019.2.3) software. Plasmids referring to several publicly published *S.* Indiana sequences were selected for genomic structure comparison against the three plasmids of *S.* Indiana CFSA664 in this study, and map generation was performed by BRIG (v0.95). Genetic environments were analyzed and displayed using Easyfig (v2.2.2).

### Plasmid conjugal transfer

In line with a previous study (Zhang et al., 2021), the transferability and frequency of colistin resistance were investigated by broth mating conjugation experiments with plasmid-free and sodium azide-resistant *E. coli* J53 as the recipient strain. The transconjugants were selected on MacConkey agar plates (Beijing Landbridge, China) supplemented with 100 mg/L sodium azide (Sigma-Aldrich) and 2 mg/L colistin (Sigma-Aldrich). Two different conjugation temperatures (30°C and 37°C) were used for the transfer of *mcr-1*-carrying plasmid in this study. Transfer frequencies were calculated as the number of transconjugants after confirmation by PCR. The colistin MIC values of the *E. coli* J53 and transconjugants were tested according to the description above.

## Results

### Antimicrobial resistance phenotypes of 224 isolates to 14 tested compounds

The percentages related to AMR for all 244 CIP-CTX co-resistant *S.* Indiana recovered from poultry are shown in Table 1 and Supplementary Table 1. All expressed resistance to at least four categories of antimicrobial compounds. The resistance rates were divided into three classes: (1) higher than 78%: CIP, CTX, NAL, AMP, CHL, SAM, FFC, GEN, SXT, and TET; (2) 31.3%: CAZ; (3) lower than 2%: CT, IMP, and MEM. No isolate was found to be resistant to carbapenem-type compounds (IMP and MEM). Minor resistance differences were observed between *S.* Indiana isolated from different provinces, with the exception of CAZ and CT. Isolates from different regions showed a CAZ-resistant rate ranging from 11.1% (Beijing) to 54.8% (Jiangsu), and one colistin-resistant strain recovered from Jiangsu province, CFSA664, was identified and designated as a CIP-CTX-CT co-resistant isolate. *Klebsiella pneumoniae* ATCC™700,603 showed a 3 twofold concentration MIC value decrease for CTX tested in combination with clavulanate vs. when tested alone, the ESBL confirmation for all tested isolates was acceptable. All except seven isolates (3.1%, 7/224) from Beijing were confirmed as ESBL producers.

**Table 1:** A table showing the antimicrobial resistance profiles of 224 CIP-CTX co-resistant *Salmonella* Indiana against a panel of 14 antimicrobial agents including resistant isolate numbers and rates.

Antimicrobial drugs		Region/Province (number of isolates/%)					
Agent classes	Agent (abbreviation)	Beijing (*n* = 99)	Jilin (*n* = 60)	Jiangsu (*n* = 42)	Shaanxi (*n* = 16)	Guangdong (*n* = 7)	Total (*n* = 224)
Carbapenems	Imipenem (IMP)	0 (0.0)	0 (0.0)	0 (0.0)	0 (0.0)	0 (0.0)	0 (0.0)
	Meropenem (MEM)	0 (0.0)	0 (0.0)	0 (0.0)	0 (0.0)	0 (0.0)	0 (0.0)
(Fluoro)Quinolones	Nalidixic acid (NAL)	99 (100.0)	60 (100.0)	42 (100.0)	16 (100.0)	7 (100.0)	224 (100.0)
	Ciprofloxacin (CIP)	99 (100.0)	60 (100.0)	42 (100.0)	16 (100.0)	7 (100.0)	224 (100.0)
Cephalosporins	Cefotaxime (CTX)	99 (100.0)	60 (100.0)	42 (100.0)	16 (100.0)	7 (100.0)	224 (100.0)
	Ceftazidime (CAZ)	11 (11.1)	27 (45.0)	23 (54.8)	7 (43.8)	2 (28.6)	70 (31.3)
Penicillins	Ampicillin (AMP)	98 (99.0)	60 (100.0)	42 (100.0)	16 (100.0)	7 (100.0)	223 (99.6)
β-Lactam combination agents	Ampicillin-sulbactam (SAM)	93 (93.9)	57 (95.0)	42 (100.0)	16 (100.0)	7 (100.0)	215 (95.6)
Aminoglycosides	Gentamicin (GEN)	81 (81.8)	58 (96.7)	39 (92.9)	16 (100.0)	7 (100.0)	201 (89.7)
Tetracyclines	Tetracycline (TET)	83 (83.8)	40 (66.7)	29 (69.1)	16 (100.0)	7 (100.0)	175 (78.1)
Folate pathway inhibitors	Trimethoprim-sulfamethoxazole (SXT)	88 (88.9)	49 (81.7)	38 (90.5)	14 (87.5)	7 (100.0)	196 (87.5)
Phenicols	Chloramphenicol (CHL)	96 (97.0)	60 (100.0)	38 (90.5)	16 (100.0)	6 (85.7)	216 (96.4)
	Florfenicol (FFC)^a^^,^^c^	95 (97.0)	58 (96.7)	37 (88.1)	16 (100.0)	6 (85.7)	212 (94.6)
Polymyxin	Colistin (CT)^b^^,^^c^	0 (0.0)	0 (0.0)	1 (2.38)	0 (0.0)	0 (0.0)	1 (0.4)
ESBLs		92 (91.9)	60 (100.0)	42 (100.0)	16 (100.0)	7 (100.0)	217 (96.9)

a Interpretation according to the CLSI guidelines M31-A3, 2008.

b Interpretation according to EUCAST clinical breakpoints, 2018.

c Used as a feed additive in animal production.

### Antimicrobial resistance patterns of 224 isolates

The antimicrobial resistance patterns are shown in Table 2 and Supplementary Table 1 for all 224 CIP-CTX co-resistant *S.* Indiana isolates tested. There were 2(0.89%), 1(0.45%), 12 (5.36%), 81 (36.16%), 127 (56.70%), and 1(0.45%) isolates resistant to 4, 5, 6, 7, 8, and 9 classes of antimicrobial agent tested, respectively. All 224 isolates were defined as MDR (resistant to three or more classes of antimicrobial drugs) and 209 (93.3%) of them were identified as high-level MDR (resistant to seven or more classes of antimicrobials). Among which, only one isolate was identified to be co-resistant to nine in ten classes of all drugs tested, with the resistant pattern of GEN-CHL-CIP-NAL-AMP-SAM-TET-CTX-SXT-FFC-CT. In total, 26 different AMR profiles were recorded among 224 *S.* Indiana isolates. The most prevalent MDR profiles identified were GEN-CHL-CIP-NAL-AMP-SAM-TET-CTX-SXT-FFC (86/224, 38.39%), followed by GEN-CHL-CIP-NAL-AMP-SAM-TET-CAZ-CTX-SXT-FFC (38/224, 16.96%). The isolates from different regions showed some MDR difference, for instance, the isolates showed co-resistance to 4, 5, and 6 classes of drugs only recovered from Beijing, Jilin, and Jiangsu provinces, while the isolates recovered from Shaanxi (*n* = 16) and Guangdong (*n* = 7) provinces showed co-resistance to 7, 8, and 9 classes of drugs.

**Table 2:** The antimicrobial resistance pattern of 224 CIP-CTX co-resistant *S.* Indiana isolates.

Antimicrobial resistance pattern	Regions	Number (rate/%)	Co-resistant drug classes	Number (rate/%)
CIP-NAL-AMP-SAM-CTX	Jiangsu (1)	1 (0.45)	4	2 (0.89)
CIP-NAL-AMP-TET-CTX	Beijing (1)	1 (0.45)		
CHL-CIP-NAL-AMP-CAZ-CTX-SXT-FFC	Jilin (1)	1 (0.45)	5	1 (0.45)
CHL-CIP-NAL-AMP-SAM-CAZ-CTX-SXT-FFC	Jilin (1)	1 (0.45)	6	12 (5.36)
CHL-CIP-NAL-AMP-TET-CAZ-CTX-SXT-FFC	Beijing (1)	1 (0.45)		
GEN-CHL-CIP-NAL-AMP-CTX-SXT-FFC	Jilin (2)	2 (0.89)		
GEN-CHL-CIP-NAL-AMP-SAM-CTX-FFC	Beijing (4)	4 (1.79)		
GEN-CHL-CIP-NAL-AMP-TET-CTX-FFC	Beijing (1)	1 (0.45)		
GEN-CHL-CIP-NAL-TET-CAZ-CTX-SXT-FFC	Beijing (1)	1 (0.45)		
GEN-CIP-NAL-AMP-CTX-SXT-FFC	Beijing (1)	1 (0.45)		
GEN-CIP-NAL-AMP-SAM-TET-CTX	Jiangsu (1)	1 (0.45)		
CHL-CIP-NAL-AMP-SAM-TET-CTX-SXT-FFC	Beijing (16), Jiangsu (2)	18 (8.04)	7	81 (36.16)
GEN-CHL-CIP-NAL-AMP-SAM-CAZ-CTX-SXT-FFC	Jilin (8), Jiangsu (7)	15 (6.70)		
GEN-CHL-CIP-NAL-AMP-SAM-CTX-SXT	Jiangsu (1)	1 (0.45)		
GEN-CHL-CIP-NAL-AMP-SAM-CTX-SXT-FFC	Beijing (11), Jilin (8), Jiangsu (3)	22 (9.82)		
GEN-CHL-CIP-NAL-AMP-SAM-TET-CAZ-CTX	Jilin (2)	2 (0.89)		
GEN-CHL-CIP-NAL-AMP-SAM-TET-CAZ-CTX-FFC	Jilin (8), Jiangsu (1)	9 (4.02)		
GEN-CHL-CIP-NAL-AMP-SAM-TET-CTX-FFC	Beijing (5), Jiangsu (1), Jilin (1), Shaanxi (2)	9 (4.02)		
GEN-CHL-CIP-NAL-AMP-TET-CTX-SXT-FFC	Beijing (1)	1 (0.45)		
GEN-CIP-NAL-AMP-SAM-CTX-SXT-FFC	Jiangsu (1)	1 (0.45)		
GEN-CIP-NAL-AMP-SAM-TET-CTX-SXT	Beijing (1), Guangdong (1), Jiangsu (1)	3 (1.34)		
GEN-CHL-CIP-NAL-AMP-SAM-TET-CTX-SXT	Beijing (1)	1 (0.45)	8	127 (56.70)
GEN-CHL-CIP-NAL-AMP-SAM-TET-CAZ-CTX-SXT	Beijing (1), Jiangsu (1)	2 (0.89)		
GEN-CHL-CIP-NAL-AMP-SAM-TET-CAZ-CTX-SXT-FFC	Beijing (8), Guangdong (2), Jiangsu (14), Jilin (7), Shaanxi (7)	38 (16.96)		
GEN-CHL-CIP-NAL-AMP-SAM-TET-CTX-SXT-FFC	Beijing (46), Guangdong (4), Jiangsu (7), Jilin (22), Shaanxi (7)	86 (38.39)		
GEN-CHL-CIP-NAL-AMP-SAM-TET-CTX-SXT-FFC-CT	Jiangsu (1)	1 (0.45)	9	1 (0.45)

### CIP and CTX resistance-encoding genes

Antimicrobial resistance genes detected for the 224 CIP-CTX co-resistant *S.* Indiana were listed in Table 3 and Supplementary Table 1. Five types of amino acid substitution were identified in QRDRs. Among the isolates studied, 164 (73.2%, 164/224) possessed the dominant amino acid substitution in GyrA (S83F and D87N) and ParC (T57S and S80R), along with other substitutions in GyrA (S83F and D87G) and ParC (T57S and S80R) being observed in 56 isolates (25.0%, 56/224). These two allelic types accounted for 98.2% (220/224) of all CIP-CTX co-resistant *S.* Indiana isolates. Two isolates were identified with amino acid substitutions in GyrA (D87G) and ParC (T57S), while one isolate showed a substitution type of GyrA (S83F) and ParC (T57S). One isolate containing an amino acid substitution in GyrA (S83L and D87N) together with several nucleotide insertions and deletions (InDels) in the *parC* gene were also found. No amino acid change was observed for GyrB and ParE.

**Table 3:** A table recording the QRDRs^a^, PMQR^b^, and ESBLs^c^ genotypes of 224 CIP-CTX co-resistant *S.* Indiana.

QRDRs amino acid substitutions				Genes		Total number
GyrA	GyrB	ParC	ParE	PMQR genes	β-Lactamase genes	
S83F, D87N	–^d^	T57S, S80R	–	*oqxAB, aac-(6′)-Ib-cr*	*bla*_CTX-M-65_ (*n* = 131), *bla*_CTX-M-14_ (*n* = 8), N/A (*n* = 7)^f^	146
				*oqxAB, aac-(6′)-Ib-cr, qnrS*	*bla*_CTX-M-65_ (*n* = 15), *bla*_CTX-M-14_ (*n* = 1), N/A (*n* = 1)^g^	17
				*oqxAB, aac-(6′)-Ib-cr, qepA*	N/A (*n* = 1)^h^	1
S83F, D87G	–	T57S, S80R	–	*oqxAB, aac-(6′)-Ib-cr*	*bla*_CTX-M-14_ (*n* = 6), *bla*_CTX-M-27_ (*n* = 9), *bla*_CTX-M-28_ (*n* = 8), *bla*_CTX-M-65_ (*n* = 16), *bla*_CTX-M-79_ (*n* = 7), N/A (*n* = 2)^i^	48
				*oqxAB, aac-(6′)-Ib-cr, qnrS*	*bla*_CTX-M-14_ (*n* = 2), *bla*_CTX-M-27_ (*n* = 2), *bla*_CTX-M-28_ (*n* = 1)	5
				*oqxAB, aac-(6′)-Ib-cr, qepA*	*bla*_CTX-M-14_ (*n* = 2), N/A (*n* = 1)	3
D87G	–	T57S	–	*oqxAB, aac-(6′)-Ib-cr*	*bla*_CTX-M-65_ (*n* = 1)	1
				*oqxAB, aac-(6′)-Ib-cr, qnrS*	*bla*_CTX-M-65_ (*n* = 1)	1
S83F	–	T57S	–	*oqxAB, aac-(6′)-Ib-cr, qnrS, qepA*	*bla*_CTX-M-65_ (*n* = 1)	1
S83L, D87N	–	InDels^e^	–	*oqxAB, aac-(6′)-Ib-cr, qnrS*	*bla*_CTX-M-79_ (*n* = 1)	1

a QRDRs: quinolone resistance determinant regions.

b PMQR: plasmid-mediated quinolone resistance.

c ESBLs: extended-spectrum beta-lactamases.

d No substitution detected.

e InDels: Lots of nucleotide insertions and deletions were observed in *parC* gene.

f Four in seven were ESBL negative.

g This one was ESBL negative.

h This one was ESBL negative.

i One in two was ESBL negative.

PMQR-encoding genes, especially *oqxA, oqxB,* and *aac (6′)-Ib-cr,* were identified in all 224 *S.* Indiana tested, while *qnrS* and *qepA* genes were also detected in 25 (11.2%) and 5 (2.2%) isolates, respectively (Table 3 and Supplementary Table 1). One hundred and ninety-five isolates were harboring only the *oqxAB* and *aac (6′)-Ib-cr* genes without any *qnr* or *qepA* genes, which represented 87.1% (195/224) of this collection. In addition to the *oqxAB* and *aac (6′)-Ib-cr* genes, there were 24 and 4 isolates carrying *qnrS* gene and *qepA* gene separately, and one isolate was observed to carry both *qnrS* and *qepA* genes, which also possessed two point mutations in QRDRs including amino acid substitutions in GyrA (S83F) and ParC (T57S). The PMQR genes *qnrA*, *qnrB*, *qnrC,* and *qnrD* were not detected.

Among 217 ESBL positive CIP-CTX co-resistant *S.* Indiana, we identified five subtypes of *bla*_CTX-M_ gene, including *bla*_CTX-M-65_ (*n* = 165), *bla*_CTX-M-14_ (*n* = 19), *bla*_CTX-M-27_ (*n* = 11), *bla*_CTX-M-28_ (*n* = 9), and *bla*_CTX-M-79_ (*n* = 8), while five isolates were *bla*_CTX-M_ group negative. Of these, isolates with the *bla*_CTX-M-65_ gene ranked the top with a rate of 76.0% (165/217) overall. The QRDRs, PMQR, and ESBLs genotypes of 224 CIP-CTX co-resistant *S.* Indiana are shown in Table 3 and Supplementary Table 1.

### Screening for *mcr* gene-positive isolate

The complete collection was tested for the presence of the *mcr-1*–*mcr-10* genes and only one *S.* Indiana (0.45%, 1/224) denoted as CFSA664 was found to be positive for *mcr-1* without any other mcr genes being detected, and this isolate was also detected as the only isolate exhibiting CTX-CIP-CT co-resistance. This isolate was recovered from a chilled, packaged chicken sample collected from a supermarket in Yangzhou city in Jiangsu province in 2011.

### Antimicrobial susceptibility testing profile for *mcr* gene-positive isolate

The MIC values and resistance phenotypes against an extended panel of 27 antimicrobial compounds for this isolate were listed in Table 4. The isolate showed an MIC value of 4 mg/L which was a relevant low level of colistin resistance (2–8 mg/L) but was susceptible to ceftriaxone, cefepime, three carbapenem drugs, aztreonam, and tigecycline, while expressing an intermediate resistant phenotype to cefoxitin, nitrofurantoin, and polymyxin B. Further, this isolate was confirmed as an ESBL positive strain and demonstrated an MDR phenotype against ten classes (16 kinds) of antimicrobial compounds.

**Table 4:** Antimicrobial susceptibility of CIP-CAZ-CT co-resistant *S.* Indiana CFSA664 to a panel of antimicrobial agents.

Antimicrobial class	Antimicrobial agent (abbreviation)	MIC (mg/L)	R/I/S^a^	Resistance genes or point mutation	
				Chromosome	Plasmid
Penicillins	Ampicillin (AMP)	≥32	R		*bla*_OXA-1_, *bla*_CTX-M-65_, *bla*_TEM-1B_
β-Lactam combination agents	Ampicillin/sulbactam (SAM)	≥32/16	R		
Cephalosporins	Cefotaxime (CTX)	≥16	R		
	Cefotaxime+clavulanate (CTX + CLA)	0.12/4	–		
	Ceftazidime (CAZ)	≥16	R		
	Ceftazidime+clavulanate (CAZ + CLA)	0.5/4	–		
	Cephalothin (KF)	≥32	R		
	Cefoxitin (FOX)	16	I		
	Ceftriaxone (CRO)	0.25	S		
	Cefepime (FEP)	2	S		
Carbapenems	Imipenem (IMP)	0.12	S		
	Meropenem (MEM)	0.03	S		
	Ertapenem(ETP)	0.5	S		
Monobactams	Aztreonam (ATM)	0.12	S		
Aminoglycosides	Gentamicin (GEN)	≥16	R	*aac(6′)-Iaa*	*armA, aadA5, aph(6)-Id, aph(3″)-Ib, aac(3)-IV, aph(3′)-IIa, aph(4)-Ia, aac(6′)-Ib-cr*^e^
	Amikacin (AK)	≥64	R		
Tetracyclines	Tetracycline (TET)	≥16	R		*tet(A)*
	Tigecycline (TGC)	0.25	S		
(Fluoro)Quinolones	Nalidixic (NAL)	≥32	R	GyrA (S83F, D87N)	*oqxAB*^f^*, aac(6′)-Ib-cr*^e^
	Ciprofloxacin (CIP)	≥8	R		
Folate pathway inhibitors	Trimethoprim/sulfamethoxazole (SXT)	≥8/152	R		*sul1*, *sul2*^g^, *dfrA17*
	Trimethoprim (TMP)	≥16	R		
Phenicols	Chloramphenicol (CHL)	≥32	R		*floR, catB3*
	Florfenicol (FFC)^b^^,^^c^	≥16	R		
Nitrofurans	Nitrofurantoin (NIT)	64	I		
Polymyxins	Polymyxin E (Colistin, CT)^c^^,^^d^	4	R		*mcr-1*^h^
	Polymyxin B	4	I		
Fosfomycins	Fosfomycin (FOS)	≥32/16	R		*fosA3*
Rifampicin^i^	–	–	–		*arr-3*
Macrolide^i^	–	–	–		*mph(A)*
Disinfectant resistance^i^	–	–	–		*oqxAB*^f^*, qacE*△*1*

R, resistant; I, intermediate; S, susceptible.

a R/I/S according to the CLSI guidelines M100-S28, 201.

b R/I/S according to the CLSI guidelines M31-A3, 2008.

c Used as a feed additive in animal production.

d R/I/S according to EUCAST clinical breakpoints, 2018.

e *aac(6′)-Ib-cr* gene only got one copy but mediated two resistance mechanisms.

f *oqxA* and *oqxB* genes only got one copy but mediated two resistance mechanisms.

g Two *sul2* genes were detected on the same plasmid but different position.

h *mcr-1* gene was located on plasmid pCFSA664-3 while other plasmid-mediated antimicrobial resistance-coding genes localized at pCFSA664-1.

i No AST data but with antimicrobial-resistant genes predicted from genome data.

### Genome features of *Salmonella* Indiana CFSA664

Based on the genome data, CFSA664 was confirmed as *S.* Indiana and identified as the MLST type of ST17 by the SISTR platform. Sequencing provided 88,121 reads in total with a mean read length of 8,319 bp and coverage of 85.69x. There were 4,760 coding genes and 176 pseudogenes along with 120 RNA genes (22 rRNAs, 86 tRNAs and 12 ncRNAs) within this genome from the prediction of NCBI PGAP annotation, and it consisted of a single circular chromosome (4,733,813 bp, 52.1% GC content) and three circular plasmids: plasmid pCFSA664-1 (255,327 bp, 47.9% GC content), plasmid pCFSA664-2(41,696 bp, 45.4% GC content), and plasmid pCFSA664-3(61,841 bp 42.4% GC content). The replicon type of plasmid pCFSA664-1 included IncHI2, IncHI2A, IncN, and RepA-pKPC-CAV1321, presenting a multiple Inc. type in the same plasmid; plasmids pCFSA664-2 and pCFSA664-3 were denoted as IncP1 and IncI2 replicon types, respectively. The virulence factors (VFs) of CFSA664 predicted from VFDB database were shown in Supplementary Table 2.

### Resistome of *Salmonella* Indiana CFSA664

**Figure 1:**
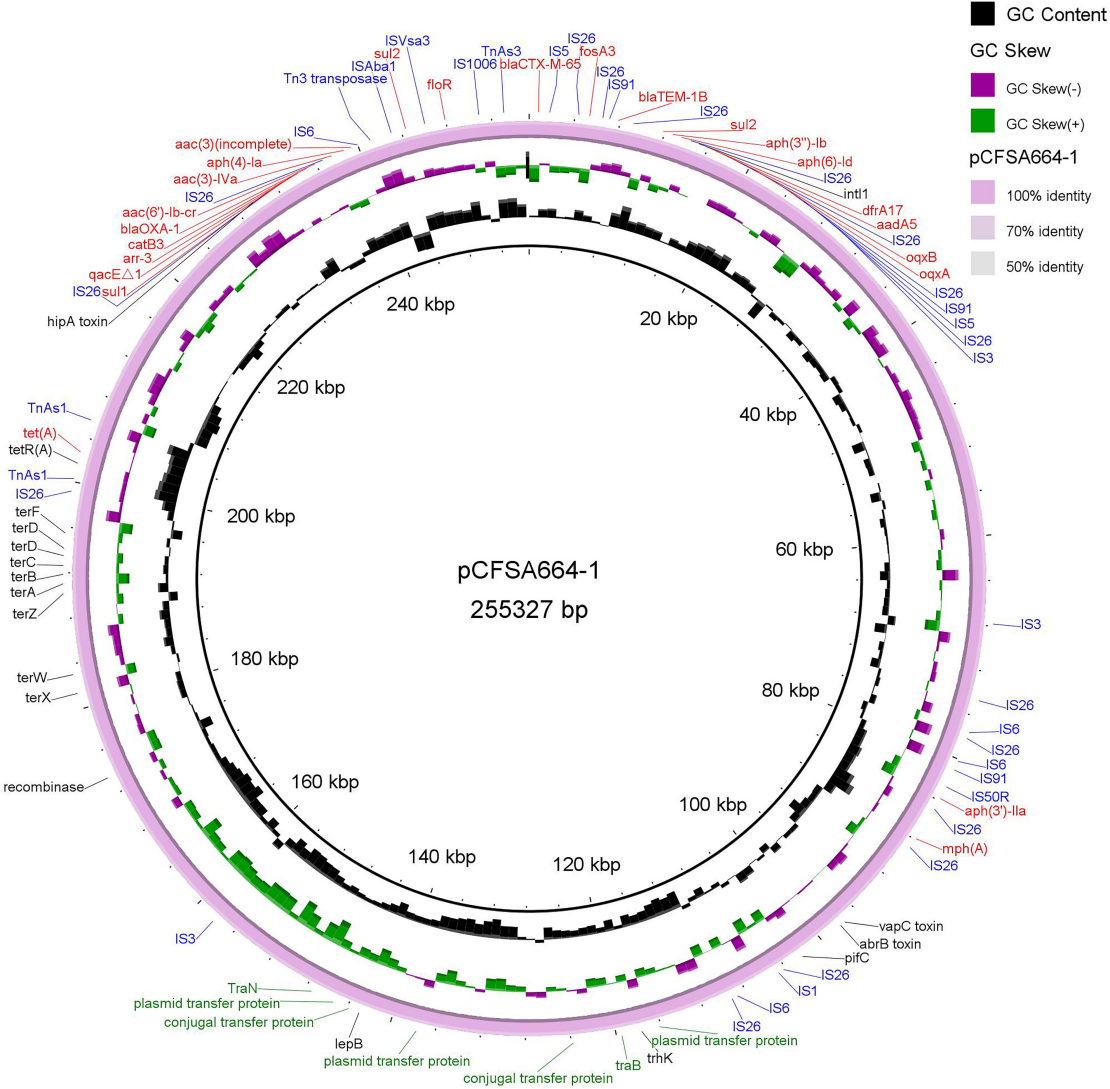
Representation of plasmid pCFSA664-1 (accession no. CP033353). Antimicrobial resistance genes (color in red), insertion sequences (color in blue), plasmid transfer protein and conjugal transfer protein (color in green), and other genes, including tellurium resistance genes, were shown in the circle, based on BRIG (BLAST Ring Image Generator) tool. Plasmid pCFSA664-1 was used as the reference genome sequence itself. Individual rings range from 1 (inner ring) to 4 (outer ring): ring 1, backbone; ring 2, GC skew of plasmid pCFSA664-1; ring 3, GC content of plasmid pCFSA664-1; ring 4, plasmid pCFSA664-1 conservation plot.

As shown in Table 4, two point mutations were identified in the *gyrA* and *parC* genes on the chromosome of *S.* Indiana CFSA664, and these gave rise to amino acid substitutions of GyrA (S83F, D87N) and ParC (T57S, S80R), mediating the quinolone resistance phenotype detected. A total of 26 antimicrobial resistance-encoding genes were identified in the genome of *S.* Indiana CFSA664, including two copies of the *sul2* gene on the same plasmid pCFSA664-1. No AMR gene was found on the IncP1-type plasmid pCFSA664-2. The *aac (6′)-Iaa* gene and the *mcr-1* gene were the only resistance genes localized to the chromosome and the IncI2 plasmid pCFSA664-3, respectively. All of the other 24 plasmid-mediated antimicrobial resistance-coding genes, including three ESBL genes (*bla*_CTX-M-65_, *bla*_OXA-1_, *bla*_TEM-1B_), were located on the same plasmid pCFSA664-1. Twenty-one of these 24 genes were mapped within an ~56-kbp locus (230,792–31,620 bp, Figure 1) that contained more than 22 mobile genetic elements (MGE) including insertion sequence (IS) elements and transposon CDSs, such as IS*3*, IS*4*, IS*5*, IS*6*, IS*91,* and Tn*3* family transposase, and also IS*6*-, IS91-and Tn*3*-like element (IS*26*, IS*1006*, IS*Vsa3,* and Tn*As3*) family transposase. Additionally, 16 ORFs and 11 hypothetical proteins were also included in this region. BLASTn comparisons indicated that a high similarity (100% coverage value and 99.99% nucleotide identity) of this region was observed mapping to a plasmid pD90-1 (GenBank no. CP022451.1) from an *S.* Indiana D90, which was isolated from a whole chicken carcass collected in a poultry slaughterhouse in 2012 in Henan, China and carrying four different Inc. type plasmids (HI2/HI2A/N/Q1-, I2-, N/X1-and unknown-Inc types). In terms of the remaining three of the 24 resistance genes [*mph* (A), *aph (3′)-IIa,* and *tet* (A)], they were separated and positioned at ~84-, 88- and 203-kbp, respectively on plasmid pCFSA664-1, with the number of transposase CDSs varying from 1 to 4 (Figure 1). Other than these AMR genes observed in plasmid pCFSA664-1, a *ter* gene operon (*ter*ABCDFWXZ), required for mediating resistance to potassium tellurite, was situated in front of *tet* (A) gene, positioning at 180–200-kbp (Figure 1).

### Structure of *mcr-1*-carrying plasmid pCFSA664-3

**Figure 2:**
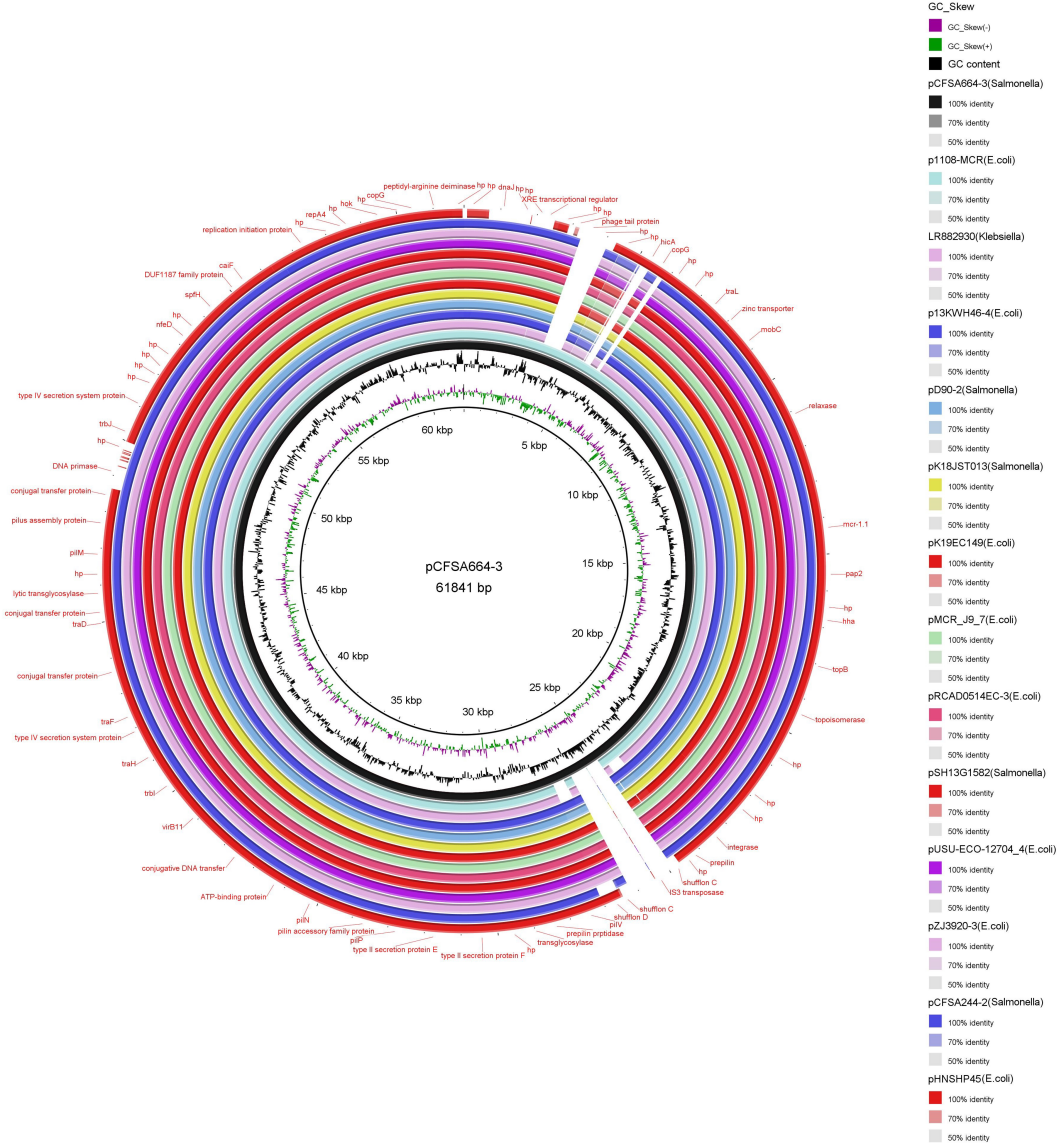
Circular comparison of the structure of plasmid pCFSA664-3 (accession no. CP033355) and thirteen Enterobacteriaceae plasmids with similar backbone structures based on BRIG (BLAST Ring Image Generator) tool. Plasmid pCFSA664-3 was used as the reference genome sequence. Individual rings range from 1 (inner ring) to 17 (outer ring): ring 1, backbone; ring 2, GC skew of plasmid pCFSA664-3 reference genome; ring 3, GC content of plasmid pCFSA664-3 reference genome; ring 4, plasmid pCFSA664-3 conservation plot; ring 5, plasmid p1108-MCR (accession no. MG825380) conservation plot; ring 6, plasmid pLR882930 (accession no. LR882930) conservation plot; ring 7, plasmid p13KWH46–4 (accession no. CP019254) conservation plot; ring 8, plasmid pD90-2 (accession no. CP022452) conservation plot; ring 9, plasmid pK18JST013 (accession no. CP065423) conservation plot; ring 10, plasmid pK19EC149 (accession no. CP050290) conservation plot; ring 11, plasmid pMCR_J9_7 (accession no. CP075067) conservation plot; ring 12, plasmid pRCAD0514EC-3 (accession no. CP034109) conservation plot; ring 13, plasmid pSH13G1582 (accession no. MH522412) conservation plot; ring 14, plasmid pUSU-ECO-12704_4 (accession no. KY657478) conservation plot; ring 15, plasmid pZJ3920-3 (accession no. CP020548) conservation plot; ring 16, plasmid pCFSA244-2 (accession no. CP033254) conservation plot; ring 17, plasmid pHNSHP45 (accession no. KP347127) conservation plot.

A total of thirteen plasmids, which were collected from Enterobacteriaceae (*E. coli*, *n* = 8; *Salmonella, n* = 4; *Klebsiella*, *n* = 1) isolates from GenBank and had an almost identical backbone structure, were used for structural comparative analysis with plasmid pCFSA664-3 (Figure 2). Plasmid pCFSA664-3 could be differentiated from these other plasmids by an insertion sequence element IS*3* transposase, a phage tail protein, and *hicA*-coding protein. In terms of the *mcr-1* gene region, all plasmids demonstrated a high degree of sequence conservation. In plasmid pCFSA664-3, the sequences relevant to shufflon C appeared to be disrupted by IS*3* transposase, a feature which was missing in all other plasmids. Plasmid pHNSHP45 (Accession number: KP347127), belonging to the first isolate reported to contain an *mcr-1* gene (*Escherichia coli* strain SHP45), was devoid of three more sequences for encoding DNA primase, DnaJ, and XRE transcriptional regulator. Several conjugal transfer proteins were observed in these plasmids, and the *mcr-1* gene was the only acquired gene conferring resistance to a known antimicrobial agent. A zinc transporter gene was also located near the *mcr-1* and relaxase locus.

### Genetic environment context of *mcr-1* gene

**Figure 3:**
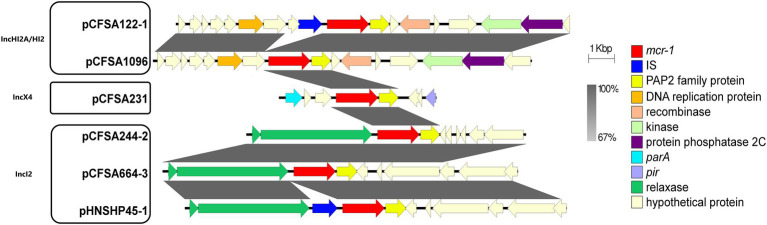
A figure showing the schematic representation of the genetic environments related to the *mcr-1* gene in various bacterial plasmids. The figure was generated by Easyfig (v2.2.2). Plasmids marked with “pCFSA” were carried by 4 *mcr-1* positive *Salmonella* isolates from our previous study and one isolate from this study, while plasmid pHNSHP45(Accession number: KP347127) belonged to *Escherichia coli* strain SHP45, which is the first isolate reported harboring *mcr-1* gene. Replicon types are shown into three groups for all plasmids. Confirmed and putative open reading frames (ORFs) are indicated by block arrows and their orientations with different colors, and arrow size is proportional to the predicted ORF length. *mcr-1* gene is indicated by a red arrow, while genes encoding mobile elements (Insertion sequence, IS) are indicated by blue arrows. Regions of homology between the plasmids ranging from 67 to 100% are indicated by the graded shaded regions between sequences.

To better understand the genetic environment of the *mcr-1* locus of plasmid pCFSA664-3, sequences in different plasmids, belonging to three replicon types, were extracted and compared (Figure 3). This analysis revealed that the *mcr-1* genes in three IncI2 type plasmids (pCFSA664-3, pHNSHP45, and pCFSA244-2) were found to be located between a PAP2 family protein-encoding gene (arrowed in yellow) and a relaxase-encoding gene (arrowed in green). In the case of plasmid pHNSHP45, an IS*30* family element IS*Apl1* was followed by its relaxase-encoding gene downstream. In comparison with these three plasmids, three other plasmids used in this study did not have a relaxase-encoding gene upstream of the *mcr-1* gene but processed some hypothetical proteins and some ORFs. In terms of plasmid pCFSA664-3 in this study, together with plasmids pCFSA231 (IncX4), pCFSA1096 (IncHI2A/HI2), and pCFSA244-2 (IncI2), the *mcr-1* genes were identified locating with a *pap2*-encoding gene distal to this site, but without any insertion sequences (ISs) coding genes, and this was different when compared with plasmids pHNSHP45 and pCFSA122-1.

### Transfer by conjugation of plasmid pCFSA664-3

In this study, plasmid pCFSA664-3, the *mcr-1*-carrying plasmid in *S.* Indiana CFSA664, could transfer into *E. coli* J53 at relatively high frequencies (1.6 × 10^−4^) per recipient (30°C) and 2.2 × 10^−4^ per recipient (37°C). This was confirmed following the selection of 5 *mcr-1*-positive transconjugants (*E. coli* J53 + pCFSA664-3) by PCR, with MIC values of 4 mg/L to colistin, similar to that described previously for CFSA664.

## Discussion

Based on the surveillance data relevant to the serotype distribution and antimicrobial resistance of *Salmonella* from chicken sourced in different regions of China in our previous study (Hu et al., 2018a), we re-characterized the isolates that were expressing resistance to both ciprofloxacin and cefotaxime, all of which were identified as serovar Indiana, suggesting that CIP-CTX co-resistant *S.* Indiana had a broad geographical distribution. High antimicrobial resistance rates (78% was the lowest) to most of the tested compounds were observed in the *S.* Indiana study collection. This is not surprising since these drugs are routinely used for the treatment of *Salmonella* infections both in the clinical setting and in food-producing animals. At the same time, this feature does not link with the original statistical description for general *S.* Indiana isolates (Gong et al., 2017). A resistant rate of 31.3% to CAZ, another important first-line third-generation cephalosporin, was observed. Only one isolate exhibited colistin resistance while none were resistant to imipenem and meropenem. Though compared with Beijing, Jilin, and Jiangsu, less CIP-CTX co-resistant *S.* Indiana isolates were obtained from Shaanxi and Guangdong provinces, they showed a distinct high level of multi-drug resistance to the compounds we tested.

Double amino acid substitutions in GyrA (S83F and D87N)/GyrA (S83F and D87G) and ParC (T57S and S80R) were noted in most of the study isolates, and this was consistent with our previous report relevant to *S.* Indiana from patients and food-producing animals in China (Bai et al., 2016). No mutations were detected in either *gyrB* or *parE*. PMQR-encoding genes of *oqxAB*, *aac-(6′)-Ib-cr* were identified among all tested strains. In contrast, fewer isolates were identified to be positive for the other two PMQR genes (*qnrS*, *n* = 25; *qepA*, *n* = 3), especially for *qepA*. Our data showed various acquisition types for transferable PMQR genes among our isolates. Our findings highlighted the fact that PMQR confers low-level quinolone resistance, but previous studies indicated these markers could facilitate the subsequent emergence of high-level resistance *via* mutation(s) in one or more of the topoisomerase genes (Hernández et al., 2011).

The subgenotypes of *bla*_CTX-M_ were regularly varying by different *Salmonella* serovars, for instance, *bla*_CTX-M-55_ in *S. enteritidis* (Li et al., 2021), *bla*_CTX-M-9_ in *S*. Kentucky (Chen et al., 2021), and *bla*_CTX-M-14_ in *S. typhimurium* (Wong et al., 2014). Seven kinds of *bla*_CTX-M_ variants were identified among 407 *S.* Indiana ST17 isolates collected from foods, patients, and environments in 16 provinces of China from 2002 to 2018, and among these variants, *bla*CTX-M-65 (*n* = 55) was the most common one in ST17 isolates, followed by *bla*_CTX-M-55_ (*n* = 14), *bla*_CTX-M-14_ (*n* = 6), *bla*_CTX-M-27_ (*n* = 6), *bla*_CTX-M-123_ (*n* = 3), *bla*_CTX-M-15_ (*n* = 1) and *bla*_CTX-M-90_ (*n* = 1). Although *bla*_CTX-M-65_ was also the most common *bla*_CTX-M_ subgenotype identified among the *S.* Indiana isolates in this study, it remains to be clarified how this event emerged, and further research on the selection, dissemination, and maintenance of MGE carrying this gene may provide some clues, as *bla*_CTX-M_ is often located on conjugative plasmids, which might contribute to the rapid expansion of ceftriaxone resistance through conjugation among Enterobacteriaceae (Zhang et al., 2022).

Three *mcr-1.1* gene positive (0.88%, 3/341) *Salmonella* were detected among 341 *Salmonella* isolated from 1,234 raw retail meat samples collected in Beijing, China, 2017 (Lyu et al., 2021). Another study, investigating Salmonella isolated from farms, downstream abattoirs, and markets of chickens in Sichuan province of China, showed a relevant higher percentage of *mcr-1* gene (8.3%, 2/24; 14.3%, 4/28; 13.9%, 5/36; Ma et al., 2017). One *S. typhimurium* cultured from RTE prepared pork was found to contain the *mcr-1* gene was identified among 30 *Salmonella* isolates (3.33%, 1/30) recovered from RTE foods collected from mainland China in 2014 (Wang et al., 2018). According to our latest data from China national food safety risk surveillance network, 18 in more than 5,100 foodborne *Salmonella* recovered from various kinds of foods collected in mainland China between 2011 and 2020 were detected as *mcr-1* positive, corresponding to 9 serotypes and accounting for a positivity rate of 0.35% (data not published), which could be regarded as a low prevalence rate (Hu et al., 2021). In this study, only one *mcr-1*-carrying isolate CFSA664 was recovered among 224 CIP-CTX co-resistant *S.* Indiana (0.45%), expressing MIC values of 4 mg/L, which were consistent with *mcr-1* mediating low-level colistin resistance (2–8 mg/L; Zhang et al., 2019).

From the whole genome data, three plasmids (pCFSA664-1~3) were detected in *S.* Indiana CFSA664. We identified a 56-kbp locus similar to a *Salmonella* Genomic Island (SGI) located on plasmid pCFSA664-1, with several different AMR genes and various insertion sequence elements or transposases. This SGI-like region might have undergone a series of unique events designed to capture and integrate resistance genes with the help of these ISs or transposases, resulting in stabilization and subsequent dissemination of these resistant genes. Three different types of *β*-lactamase were also observed at the same time in this SGI-like region, a feature that is rarely observed in a plasmid of *S.* Indiana. In terms of plasmid pCFSA664-2 and the *mcr-1*-carrying plasmid pCFSA664-3, several Enterobacteriaceae plasmids submitted to NCBI were found to share the same backbone structure, highlighting the successful spread of these plasmids among *S.* Indiana and other genera in the Enterobacteriaceae family. Our results support the fact that plasmids could contribute to the gene movement and the rapid spread of the *mcr-1* gene worldwide. This also indicated that the plasmids identified in this study might need further investigation for their transferability, the impact on bacterial cell fitness due to the maintenance of the conjugative plasmids, and how they are involved in the rapid spread of multiple antimicrobial-resistant determinants. Hence, surveillance for the emergence of these types of *S. enterica* serovar Indiana isolates should be undertaken as a routine public health protection measure (Dong and Soong, 2021).

After all, there are some limitations of this study as below: (1) The *Salmonella* Indiana isolates in this retrospective study were recovered from chicken samples collected from 2010 to 2012 and in only six provinces of China, and isolates from newly collected samples from more regions of prolonged monitoring would give more accurate results; (2) Whole genome sequencing technology would help to state the genetic evolution analysis or traceability, and to explore the phylogenomic relationship and genetic diversity among this batch of Salmonella Indiana isolates; (3) Pathogenicity data for the severe MDR CFSA664 would help with assessing the overall food safety or public health risk to humans in combination of the antimicrobial resistance data.

## Conclusion

This study described the phenotypic AMR patterns and mechanisms for ciprofloxacin and cefotaxime co-resistant *Salmonella* Indiana of retail chicken origin from China. *S.* Indiana exhibited a highly drug-resistant phenotype in our study. One *mcr-1*-carrying isolate with co-resistance to ciprofloxacin, cefotaxime, and colistin was identified. The complexity of the ESBL and MDR phenotype, AMR genes, and the transferability of the *mcr-1*-harboring plasmid demonstrated subsistent public health risk. Our study as we know, AMR One Health Strategies had already focused on the access and the use of antimicrobials in clinical for human medicine, but in food production, which is anther major area of the use for antimicrobial compounds, has not yet systematically adapted to achieve the one health objectives (George, 2019), so continuous surveillance and the One Health strategy are needed to limit the emergence of *S.* Indiana across the food chain.

## Nucleotide accession numbers

The genome data of chromosome and plasmid sequences of *S.* Indiana CFSA664 were deposited in the NCBI nucleotide database under BioProject no. PRJNA498334 with Biosample no. of SAMN10292850. Related accession numbers were: CP033356, CP033353, CP033354, and CP033355.

## Data availability statement

The datasets presented in this study can be found in online repositories. The names of the repository/repositories and accession number(s) can be found at: https://www.ncbi.nlm.nih.gov/genbank/, CP033356, CP033353, CP033354 and CP033355.

## Author contributions

YuH performed the literature search. YuH, YiH, FL, and SF designed the research. YuH, YiH, SN, CL (4th author), XG, WW, YD, CL (5th author), and JX performed the experiments and collected the data. YuH, YiH, SN, CL (4th author), WW, and JX analyzed and interpreted the data and finished the figures and tables. YuH, YiH, SN, and CL (4th author) wrote the manuscript. FL and SF reviewed and edited the manuscript. YuH, YiH, SN, CL (4th author), FL, and SF have accessed and verified the underlying data. All authors contributed to the article and approved the submitted version.

## Funding

This work was supported financially by the National Key Research and Development Program of China (2018YFE0101500 and 2020YFF0305001). The sponsors of the study had not any role in the study design, data collection, data analysis, interpretation, and writing of the report. We have not been paid to write this article by a pharmaceutical company or other agency. We state that all authors had full access to the full data in the study and accept responsibility to submit for publication.

## Conflict of interest

The authors declare that the research was conducted in the absence of any commercial or financial relationships that could be construed as a potential conflict of interest.

## Publisher’s note

All claims expressed in this article are solely those of the authors and do not necessarily represent those of their affiliated organizations, or those of the publisher, the editors and the reviewers. Any product that may be evaluated in this article, or claim that may be made by its manufacturer, is not guaranteed or endorsed by the publisher.
